# Knowledge, attitude, and practices of tsunami-prone communities, Nias, Indonesia

**DOI:** 10.4102/jamba.v16i1.1639

**Published:** 2024-09-09

**Authors:** Furqan I. Aksa, Muhammad Ashar, Heni W. Siswanto

**Affiliations:** 1 Department of Geography Education, Faculty of Education, Universitas Samudra, Langsa, Indonesia; 2 Department of Informatic Engineering, Faculty of Engineering, Universitas Negeri Malang, Malang, Indonesia; 3 Center for Educational Research, Social Sciences and Humanities Research Organization, National Research and Innovation Agency (BRIN) Republic of Indonesia, Jakarta, Indonesia

**Keywords:** knowledge, attitude, practices, tsunami, preparedness

## Abstract

Nias Island is presently being confronted with a substantial and imminent threat of a tsunami. However, the level of knowledge of the communities on Nias Island regarding earthquake and tsunami hazards is limited. This study aims to evaluate the level of knowledge, attitudes, and practices (KAP) among the communities residing on Nias Island with respect to the hazard of tsunami. This study adopted a community participation method derived from a social inductive study. Data were collected from field observations and in-depth interviews with 210 communities. Data were analysed using a descriptive table form for comprehensive examination. The results showed that the majority of communities lacked comprehensive knowledge regarding both the sources of tsunamis and estimated times of arrival (ETAs) for tsunamis. The findings also indicated a lack of regular disaster evacuation drills or simulations conducted by the government in the area. Moreover, there is a prevailing belief among the communities that God will prevent loss of life in the event of a tsunami. This belief is concerning because of its potential contribution to a fatalistic attitude towards disasters, potentially resulting in inadequate preparedness. The findings of this research confirm previous research that found that people living on small islands in Indonesia have very limited knowledge of the tsunami hazard.

**Contribution:**

These findings highlight the important role of the government to carry out substantial efforts aimed at enhancing the resilience of communities residing in small islands. Currently, small islands receive less attention in efforts to reduce disaster risk.

## Introduction

Nias Island is located within a seismic and tsunami-prone region that is vulnerable to frequent seismic activities and the potential occurrence of devastating tsunamis (Harada, Shoji & Takafuji 2023). Geologically, the island is located in the Sunda Subduction Zone, making it highly susceptible to active seismic activity (Syamsidik et al. 2020). Nias is ranked sixth among the most earthquake-prone areas in the world, and this region is one of the poorest and most remote in North Sumatra Province (Guarnacci 2012). Accordingly, some of the most severe earthquake and tsunami disasters in the area occurred on 26 December 2004, and 28 March 2005 (Harada et al. 2023). The 2005 earthquake, with a magnitude of Mw 8.7 on the Richter scale, resulted in 839 deaths and over 70 000 people losing their homes (Guarnacci 2012).

According to Philibosian et al. (2016), there is an anticipated likelihood of earthquake and tsunami disasters recurring in the area (Philibosian et al. 2016). This prediction was made considering the fact that Nias Island is part of the highly active Andaman–Nicobar–Sumatra (ANS) zone. As stated, this area is characterised by high pressure and the potential for a large-scale earthquake exceeding ≥ 8.0 within the next 50–100 years (Mishra et al. 2021).

In order to reduce the risk of tsunamis, it is essential to improve the knowledge, attitudes, and practices (KAP) of communities in effectively confronting this natural hazard. Knowledge is a crucial aspect of disaster risk reduction (Weichselgartner & Pigeon 2015). This is primarily because comprehensive knowledge has been observed to foster the making of effective decisions that are tailored towards the specific goal of saving lives during a disaster (Aksa et al. 2020a). Furthermore, referring to the Protective Action Decision Model (PADM) theory developed by Lindell and Perry (2012), knowledge significantly influences the decision-making processes of communities during disaster occurrences (Lindell & Perry 2012).

According to Kanhai et al. (2016), the attitudes possessed by communities towards tsunamis are intertwined with their beliefs concerning the likelihood and consequences of future disasters (Kanhai et al. 2016). This understanding underscored the importance of attitude assessment, as it helps in comprehending and analysing human behaviours when confronted with disasters (Muzenda-Mudavanhu, Manyena & Collins 2016; Naseri & Kang 2017). Following this, tsunami-related practices involve the actions carried out by communities before, during, and after the occurrence of the disaster (Kanhai et al. 2016; Salah & Sasaki 2021; Sinha et al. 2008). These practices are crucial, especially for communities residing in hazard-prone areas. This is primarily because the practices aid in the provision of information regarding the capability of communities to understand signs and take appropriate actions before a tsunami occurs.

However, data concerning the level of understanding possessed by the communities on Nias Island regarding earthquake and tsunami hazards are still limited. It has been observed that small islands usually receive insufficient attention in the context of disaster management (Shultz et al. 2016). This was primarily attributed to their remote characteristics, limited infrastructure, as well as social and economic disparities compared to mainland areas or larger cities (Shultz et al. 2016).

This study aims to evaluate the level of KAP among the communities residing on Nias Island pertaining to the hazards associated with tsunamis. In this regard, surveys carried out with a primary focus on KAP can serve as valuable tools for assessing the awareness level (knowledge), feeling (attitude), and activities (practices) that can be carried out by communities to reduce the impact of a tsunami (Kanhai et al. 2016; Sinha et al. 2008).

This research is very important to carry out because KAP are important components in disaster risk reduction. Knowledge is directly related to attitude and practice (Salah & Sasaki 2021). Knowledge, attitudes, and practices studies can be useful tools in the evaluation of existing tsunami awareness and preparedness in the small island.

## Study area

This study was carried out between July and August 2023 within coastal communities situated on Nias Island. The area of Nias Regency is 853.44 km^2^ surrounded by the Indian Ocean. The geographical features in Nias include mountains with a height above sea level between 0 and 800 m. The population of Nias Island is 147 794 people.

**FIGURE 1:**
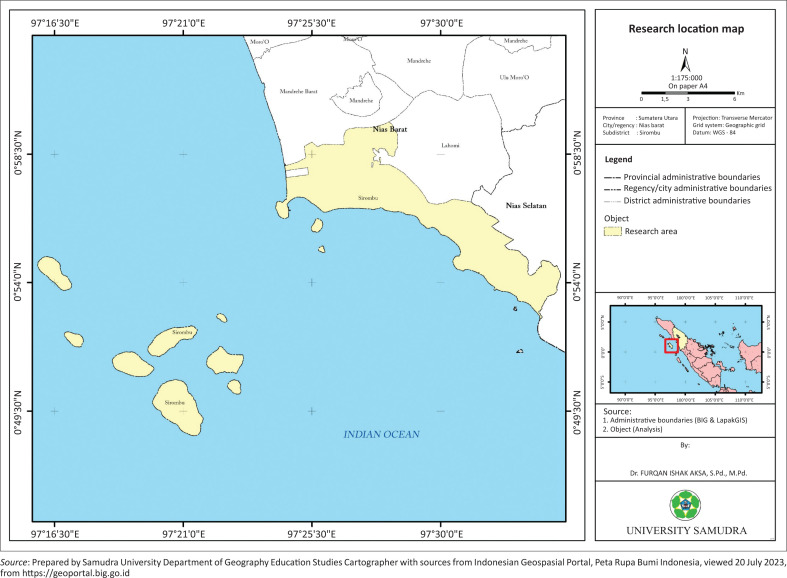
The study area.

This island comprised four distinct regions, namely North Nias, Gunung Sitoli, South Nias, and West Nias. The investigation focused primarily on the West Nias Regency, specifically the Sirombu sub-district see figure 1. This area was selected as the point of interest mainly because it was most affected by the 2004 tsunami, resulting in the tragic loss of over 200 lives. Accordingly, West Nias is situated on the western coast of Sumatra, directly borders the Indian Ocean, and hosts a population of approximately 13 622 individuals. Sirombu sub-district is an earthquake and tsunami prone area. However, so far, a survey of community KAP in dealing with the tsunami disaster has never been carried out in this area.

## Research methods and design

In order to effectively evaluate the understanding level, attitudes, and behaviours of the communities situated on Nias Island concerning tsunami hazards, this study utilised in-depth interviews and comprehensive observations. Accordingly, the selection of the study site, the Sirombu sub-district, was based on its direct coastal adjacency to the Indian Ocean and the significant impact of the 2004 tsunami on the area.

This study adopted a community participation method derived from a social inductive study, resulting in descriptive results (Marfai, Sekaranom & Ward 2015). Accordingly, as explained by Kumar (2002), the community participation method could be built from interviews, observations, and Venn diagrams. Interviews, in this context, were conducted to identify what the communities know (knowledge) about tsunami hazards, their feelings (attitude) regarding the risks, and what measures have been developed (practices) to mitigate the impact.

The main contents of the questionnaire were derived from a previous study conducted by Kanhai et al. (2016). The instrument was chosen because Sirombu sub-district has the same geographical characteristics as the research. These contents consist of KAP components of communities towards tsunami hazards. The knowledge segment comprised inquiries concerning various aspects such as knowledge about tsunami sources, historical events on Nias, estimated arrival times, and the existence of disaster education programmes within communities.

The attitude component includes questions regarding the perceptions of the communities concerning the probability of a tsunami striking Nias within the next decade, their stance pertaining to an early warning system, and the significance these communities attribute to having an evacuation plan in place. Meanwhile, the practice section comprises queries about the suitable actions to be carried out prior to the occurrence of a tsunami.

In this study, the respondents were selected to represent households using purposive random sampling. The selection criteria include several factors such as literacy, age (>17 years), and native residency within the Sirombo sub-district, amounting to a total sample size of 210 individuals. Furthermore, the data gathered from the interview were analysed using descriptive tabular forms for comprehensive examination.

## Results

### Demographics

Table 1 shows the demographic characteristics of the 210 respondents interviewed in this study. The majority were male (62.8%), aged 40–49 years (52.8%), and had a senior high school education (42.8%). The demographic distribution of respondents covered the geographic areas, namely Tugalagawu village, Fadoro village, Sisobandrao village, Tetohosi village, and Hilimberua village. These villages are located on the Sirombu coastline which has a high tsunami risk.

**TABLE 1:** Demographic characteristics of respondents.

Demographic characteristics	Number	%
**Gender**		
Male	132	62.8
Female	78	37.2
**Age group (years)**		
20–29	36	17.2
30–39	63	30.0
40–49	111	52.8
**Education level**		
Primary	68	32.4
Senior High School	90	42.8
Undergraduate Programme	52	24.8

### Knowledge about tsunami

The results of the interview showed that the majority of the observed communities (85.2%) were aware of the past occurrence of a tsunami on Nias Island. Furthermore, regarding knowledge about tsunami sources, 44.3% of the respondents stated that earthquakes constitute the major reason for the occurrence of tsunamis, 35.7% mentioned underwater landslides, 20% indicated volcanic eruption, and 21% had no idea. From the obtained responses, most of the communities believed that a tsunami is caused only by an earthquake. This finding supports prior observations from several studies indicating that a considerable portion of coastal communities in Indonesia lack awareness regarding the potential of underwater landslides and volcanic activity to trigger tsunamis (Mardiatno et al. 2017; Muhari et al. 2019; Takabatake et al. 2019). For instance, many communities have been observed to possess the belief that the 2018 Sunda Strait tsunami disaster was solely caused by an earthquake. However, other communities along the Tanjung Lesung coast were surprised by the tsunami as they did not feel the earthquake (Mardiatno et al. 2017; Muhari et al. 2019; Takabatake et al. 2019).

Pertaining to tsunami signs, the information gathered indicated that most of the communities were well-informed (Table 2). In this regard, the majority recognised signs such as strong shaking during an earthquake making it difficult to stand, receding seawater, and the appearance of large waves on the horizon. However, it is important to acknowledge the fact that a significant portion of the observed communities (80.4%) were unaware of the time it takes for a tsunami to reach Nias Island if caused by local and regional sources. Only 9.1% stated a tsunami arrival time of <1 h, 6.2% between 1 and 5 h, and 4.3% over 5 h. According to numerical simulations conducted by Syamsidik et al. (2020) regarding Tsunami Estimated Times of Arrival (ETAs) on Nias Island, especially in Gunung Sitolusi, Lahewa, and Teluk Dalam, the ETAs range from 20 to 40 min with evacuation time estimates of 20–30 min (Syamsidik et al. 2020)

**TABLE 2:** Interview results on Nias Island.

Questionnaire variable	Number	%
**What are the sources of a tsunami?†**		
Seismic (earthquake – local, regional)	93	44.3
Seismic-related (landslide – local, regional)	75	35.7
Non-seismic (eruption of volcano – local, regional)	42	20.0
Do not know	44	21.0
**What are the potentially destructive impacts of a tsunami?** ‡		
Loss of life	132	62.8
Infrastructure destroyed	92	43.8
Environment destroyed	85	40.4
Do not know	27	13.0
**What are the signs or symptoms of a tsunami?** ‡		
Strong shaking during an earthquake makes individuals difficult to stand	85	40.4
Receding seawater	143	68.0
The appearance of large waves on the horizon	23	11.0
Loud sounds resembling explosions	17	8.1
**Do you know that a tsunami has occurred in Sirombu?**		
Yes	168	80.0
No	42	20.0
**Do you know whether a tsunami has occurred in other parts of the world?**		
Yes	179	85.2
No	31	14.8
**How long would it take for a tsunami to arrive on Nias if caused by local and regional sources?**		
<1 h	19	9.1
Between 1 and 5 h	13	6.2
>5 h	9	4.3
Do not know	169	80.4
**Have any public education programmes regarding a tsunami been carried out in your community?**		
Yes	7	3.3
No	176	83.8
Do not know	27	12.9

† , Respondents can select multiple responses.

‡ , The percentage is more than 100% because the respondents can select multiple responses.

Knowledge about ETAs is crucial for communities in tsunami-prone areas. This knowledge primarily aids these communities in swift evacuations, thereby mitigating the potential loss of life (Syamsidik, Rasyif & Kato 2015), especially in areas like Nias Island where options for evacuation are limited because of its geographical conditions.

The lack of knowledge about ETAs among the majority of the observed communities, as presented in Table 2, was suspected to be because of the absence of disaster education programmes conducted by the government in the areas. Following this, according to the survey results, 83.8% of the communities reported the absence of any disaster education programmes being conducted on Nias Island. This observation is very concerning, especially considering the fact that the island has a very high vulnerability index in terms of social, economic, and physical aspects compared to other disaster-prone areas in Indonesia (Guarnacci 2012). Additionally, the increasing population growth on this island can lead to a higher risk of disaster impact.

The findings of this research confirm previous research that found that people living on small islands in Indonesia have very limited knowledge of the tsunamis hazard (Syamsidik et al. 2020). Therefore, serious efforts are needed to increase community knowledge of the tsunamis hazard. This is very important to do because knowledge is the main component in reducing disaster risk (Aksa et al. 2020; Setten & Lein 2019; Weichselgartner & Pigeon 2015). Disaster knowledge also has a significant influence on disaster awareness and preparedness (Aksa 2021; Aksa et al. 2020b).

### Attitude about tsunami

The results obtained from the interviews conducted at the various communities on Nias Island showed that 46.2% of the respondents anticipate a potential tsunami impacting the island within the next 10 years. Additionally, a substantial majority (75.2%) acknowledged the risk of being affected by such a disaster. An overwhelming percentage (91%) also recognised the critical importance of an early warning system in mitigating casualties before the occurrence of a tsunami.

Considering the roles in minimising loss of life during a tsunami, a significant portion of the communities (60.4%) expressed a belief that divine intervention by God is likely to prevent fatalities. Furthermore, a substantial percentage (34.8%) of respondents believed in the ability of the government to prevent loss of life in the event of a tsunami on Nias Island. Particularly, this belief contrasted starkly with the minimal trust (3.9%) placed in individuals to perform the same role. Based on this observation, it can be seen that the level of trust given by the respondents to external factors such as God and the government, was greater than their belief in the roles of individuals to mitigate the adverse effects of disasters.

As elucidated by Xue et al. (2014), this condition is particularly concerning because the high reliance on external factors reduces the inclination of individuals to acknowledge the associated risks (Xue et al. 2014). Moreover, the belief in the intervention of God during a disaster may further augment their fatalistic attitude (Aksa 2020; Baytiyeh & Naja 2014; Xue et al. 2014). According to Rashwan and Jenkins (2017), fatalism can be defined as the belief that significant events in life are beyond the control of individuals and those adhering to this conviction believe in natural forces or luck (Rashwan & Jenkins 2017). In this regard, it is important to comprehend that high levels of fatalism attitude will reduce the preparedness of communities for disasters (Aksa 2020; Baytiyeh & Naja 2016; DeYoung et al. 2019).

The interview results, as presented in Table 3, also showed that most of the communities (64.8%) believe authorities are unprepared to mitigate the impact of a tsunami on Nias. Furthermore, field observations indicated that physical facilities such as a tsunami warning siren tower, evacuation signs, and evacuation buildings were not found on Nias Island. The absence of evacuation buildings, in this situation, can lead to a higher loss of life in the event of a tsunami as the island has short ETAs and insufficient evacuation time (Syamsidik et al. 2020). These findings indicate that the role of the government has not been optimally executed in reducing the risk associated with the occurrence of a tsunami at Nias Island.

**TABLE 3:** Attitude about tsunami.

Questionnaire variable	Number	%
**How likely do you think a tsunami will hit Nias Island in the next 10 years?**		
Very	97	46.2
Somewhat	42	20.0
Not Very	50	23.8
Not at all	21	10.0
**Do you think you will be at risk from a tsunami?**		
Yes	158	75.2
Maybe	44	21.0
No	8	3.8
**If a tsunami hits Nias, whose actions are most likely to prevent loss of life?**		
God	127	60.4
Government	73	34.8
NGOs	2	0.9
Individuals	8	3.9
**If a tsunami occurs on Nias, coastal communities and beach visitors will have enough time to self-evacuate from coastal areas. To what extent do you agree or disagree?**		
Strongly agree	4	1.9
Agree	10	4.8
Neutral	6	2.8
Disagree	52	24.7
Strongly disagree	138	65.8
**A tsunami early warning system is very important. To what extent do you agree or disagree?**		
Strongly agree	191	91.0
Agree	10	4.7
Neutral	5	2.5
Disagree	2	0.9
Strongly disagree	2	0.9
**An evacuation plan is important for coastal communities at risk from a tsunami. To what extent do you agree or disagree?**		
Strongly agree	175	83.4
Agree	27	12.9
Neutral	4	1.9
Disagree	2	0.9
Strongly disagree	2	0.9
**Local authorities are well prepared to respond to the tsunami threat on Nias. To what extent do you agree or disagree?**		
Strongly agree	2	0.9
Agree	2	0.9
Neutral	12	5.7
Disagree	58	27.7
Strongly disagree	136	64.8

NGO, non-governmental organisation.

## Discussion

### Practices about tsunami

The majority of communities interviewed (77.6%) expressed that specific natural signs like a receding coastline, turbulent water, unusual animal behaviours, and roaring sounds from the sea, would serve as indicators to promptly vacate the beach. Approximately 21% indicated a tendency to wait and observe the situation, whereas 77.1% mentioned immediate evacuation to higher ground if confronted with a strong earthquake lasting over 20 s. Additionally, 65.7% of the respondents expressed a willingness to self-evacuate (depart from the beach and move to higher ground) upon receiving a government-issued tsunami early warning.

The heightened awareness and preparedness exhibited by these communities concerning pre-tsunami practices likely stemmed from their direct experiences during the 2004 tsunami and the 2005 earthquake. This discovery is in line with earlier studies where it has been indicated that firsthand disaster encounters significantly influence the readiness of communities (Becker et al. 2017; Made et al. 2020; Sharma & Patt 2012).

The interview results showed that 30% of the communities mentioned their inclination to await government-issued evacuation orders. This tendency likely stemmed from their strong reliance on both the government and the early warning system. Accordingly, this dependency on the early warning system fosters a perception of security among the communities, which, in turn, delays immediate self-evacuation before an impending tsunami.

A similar situation occurred in Indonesia during the earthquake and tsunami in Palu on 28 September 2018. During this incident, 82.5% of the communities did not self-evacuate because of the malfunction of the early warning system in that area (Harnantyari et al. 2020). As observed, the majority of the communities waited for evacuation orders from the government, and this resulted in a significant loss of lives (Harnantyari et al. 2020). The earthquake and tsunami caused 4340 deaths, 10 679 injuries, and approximately 200 000 individuals losing their homes (Omira et al. 2019).

Table 4 presents the results of the interview conducted with respect to the practices about tsunamis. The findings indicated that the majority of communities (72.9%) on Nias Island lacked an emergency plan for a tsunami. The absence of an emergency plan, in this regard, refers to a critical deficiency in the preparedness of the community to address potential tsunami threats. As shown in the results, only 27.1% have devised an emergency plan, including the identification of evacuation points and the implementation of practice drills.

**TABLE 4:** Practices about tsunamis.

Questionnaire variable	Number	%
**If the following events occur (receding coastline, turbulent water, hearing loud roaring sounds from the sea, unusual animal movements), what would you most likely do?**		
Immediately leave and move to higher ground	163	77.6
Wait to see what happens	44	21.0
Nothing	3	1.4
**If you experience a strong earthquake (lasting more than 20 s and you cannot stand) and are on the beach, what would you most likely do?**		
Immediately leave and move to higher ground	162	77.1
Wait to see what happens	38	18.1
Nothing	10	4.8
**If you are on a fishing boat or cruise ship and feel a strong earthquake (lasting more than 20 s, and you cannot remain standing), what would you most likely do?**		
Head for deeper waters	58	27.6
Head to the shore	99	47.1
Wait to see what happens	48	22.9
Nothing	5	2.4
**If a tsunami warning is issued for Nias, which of the following actions would you take?**		
Wait for the authorities	63	30.0
Self-evacuate (leave the beach, move to higher ground)	138	65.7
Remain at the coast to view	6	2.9
Nothing	3	1.4
**Does your family have an emergency plan for a tsunami?**		
Yes	57	27.1
No	153	72.9
**Have the authorities ever carried out a tsunami evacuation simulation?**		
Yes	203	96.7
No	7	3.3

The majority of the communities (96.7%) also stated that authorities have never conducted tsunami evacuation simulations in the area. This finding confirms a previous study by Syamsidik et al. (2020), where it was indicated that routine evacuation drills or simulations were not being conducted on small islands in Sumatra, including Nias. The probable cause for this circumstance likely stems from constraints in human resources and budgetary limitations regarding the execution of evacuation simulations. This challenge was particularly substantial after the completion of programmes conducted by the Aceh-Nias Rehabilitation and Reconstruction Agency (BRR Aceh-Nias). According to Syamsidik et al. (2021), the frequency and involvement of authorities in disaster mitigation and preparedness have significantly decreased since 2012 after the 2004 Indian Ocean tsunami in Aceh, Indonesia.

Based on the findings, it was found that the KAP of the community towards the hazard of tsunamis are very low. This is because there is no disaster education programme carried out in the area. In addition, there is limited tsunami-evacuation building and there is no disaster evacuation training programme carried out routinely. The absence of tsunami evacuation simulations has been observed to reduce the capacity of communities to face disasters. On the other hand, consistent drills and simulations significantly bolster preparedness in managing and responding to a tsunami. For instance, during the earthquake and tsunami in Japan on 11 March 2011, 2900 student lives in the coastal city of Kamaishi were successfully saved (Sakurai et al. 2018). This story is known as *The Miracle of Kamaishi*. While this phenomenon may be perceived as miraculous, it fundamentally stems from an extensive process of disaster preparedness education, alongside regular drills and simulations diligently carried out by authorities (Sakurai et al. 2018).

## Conclusion

In conclusion, this study was conducted with the aim of assessing the KAP of communities on Nias Island regarding tsunami hazards. The obtained results showed that the majority of the observed communities lacked comprehensive knowledge regarding tsunami sources and many believed that the disaster was solely caused by an earthquake. Furthermore, these communities were found to be unfamiliar with the tsunami ETAs of their residential region. In accordance with this, a prevailing belief was also observed among the communities, suggesting that God is likely to prevent the loss of life in the event of a tsunami. This belief is especially concerning as it may foster a fatalistic attitude towards disasters, thereby potentially diminishing the preparedness of both individuals and the community. This attitude was evidenced by the absence of an emergency plan among the observed communities. From the obtained results, it was also found that the government has not been conducting regular tsunami evacuation drills or simulations on the island. Lastly, field observations showed the absence of physical facilities such as a tsunami warning siren tower, evacuation signs, and evacuation buildings.

The lack of tsunami evacuation building increases the risk of the number of victims if a tsunami disaster occurs in the area. Horizontal evacuation could not be carried out because of the tsunami’s rapid arrival time, geographical conditions, and delayed departure time. Tsunami Vertical-Evacuation (TVE) can save numerous human lives when horizontal evacuation is not feasible (León et al. 2023; McCaughey et al. 2017). This research suggests that the government build a tsunami evacuation building on Nias Island and increase the community’s capacity by conducting tsunami drills every year.

This research has limitations in terms of the methodology as it uses descriptive analysis. Future research needs to assess the influence of knowledge and attitudes on community preparedness in facing tsunami disasters. Future research also needs to explore factors that influence people’s attitudes, such as past experiences, culture, and religious beliefs.
